# Electronic health records-integrated mobile health interventions in primary care to improve hypertension management in Black/African American populations: a systematic review

**DOI:** 10.1093/oodh/oqaf029

**Published:** 2025-11-03

**Authors:** Emmanuel Adediran, Karen Schliep, Tess Deatley, Elena R Gardner, Kelly Baron, Bernadette Kiraly, Dominik Ose

**Affiliations:** Meharry Medical College, Department of Surgery, Nashville, TN 37208, United States; University of Utah, Department of Family and Preventive Medicine, 310 Wakara Way, Salt Lake City, UT 84108, United States; University of Utah, Department of Family and Preventive Medicine, 310 Wakara Way, Salt Lake City, UT 84108, United States; University of Utah, Department of Family and Preventive Medicine, 310 Wakara Way, Salt Lake City, UT 84108, United States; University of Utah, Department of Family and Preventive Medicine, 310 Wakara Way, Salt Lake City, UT 84108, United States; University of Utah, Department of Family and Preventive Medicine, 310 Wakara Way, Salt Lake City, UT 84108, United States; University of Utah, Department of Family and Preventive Medicine, 310 Wakara Way, Salt Lake City, UT 84108, United States; Westsächsische Hochschule Zwickau, Department of Health and Healthcare Sciences, Zwickau, Saxony, 08056, Germany

**Keywords:** primary care, hypertension, Black/African American, mobile health, electronic health records

## Abstract

Black/African American individuals face twice the risk of hypertension compared to non-Hispanic Whites in the United States (US). A synergistic strategy integrating primary care with Electronic Health Records (EHR) and mobile health (mHealth) technologies may help address hypertension disparities. This unique strategy prioritizes early disease identification, improved care access, and self-management. However, limited research exists on how primary care can effectively integrate these components to reduce hypertension in Black/African American individuals. We aimed to (i) identify characteristics of Black/African American populations represented in EHR-driven mHealth interventions for hypertension management in primary care settings and (ii) identify the specific EHR-driven mHealth interventions. We used the PubMed, Web of Science, and Scopus databases to identify studies conducted in the US. The included articles focused on (i) Black/African American individuals, (ii) EHR and mHealth integration; (iii) primary care setting, and (iv) hypertension or blood pressure as the outcome. We screened 749 studies and synthesized 14. Combined, Black/African American patients were female (61%), 52.6 years on average, with diabetes health comorbidity (35%). Included studies utilized health monitoring devices (*n* = 14), smartphone applications (*n* = 11), interactive messaging (*n* = 8), and patient portals (*n* = 3). These tools facilitated health monitoring (*n* = 14), social networking (*n* = 3), and lifestyle counseling (*n* = 14). EHR-mHealth technologies in primary care show promise to improve hypertension management in Black/African American populations. Current interventions focus on enhancing health education access, continuous health monitoring, patient–physician communication, and social engagement. Further research is needed to optimize integration into clinical workflow for hypertension management.

## INTRODUCTION

Hypertension is a persistent chronic health condition in the United States (US), and a leading contributor to severe health complications, including stroke, sudden cardiac death, hemorrhage, and mortality [1]. Concerningly, hypertension burdens are disproportionately high among racial and ethnic minority populations (e.g. Hispanic ethnicity, Black/African American, Asian, American Indian, and Native Hawaiian race). Black/African American individuals, in particular, experience 45% higher hypertension prevalence than any other racial and ethnic minority populations in the US [2]. They are also twice as likely to experience severe outcomes like stroke mortality, heart failure, and cardiovascular disease (CVD) mortality [3, 4].

Primary care systems and electronic health records (EHR) are underutilized strategies for reducing hypertension disparities [5, 6]. Primary care is in a unique position to address and prevent these severe outcomes due to its emphasis on affordability, accessibility, patient-centeredness, and continuity of care [6]. EHRs offer a powerful tool for, for example, aggregating routine clinical and patient data. This data, in turn, can assist primary care physicians in identifying high-risk patients for targeted outreach and making informed decisions on initiating and intensifying clinical interventions [7].

Additionally, increasing evidence highlights the importance of EHR’s in hypertension management [5, 8, 9]. Our prior review found that the EHR can be used to identify high-risk patients with hypertension, tailor interventions, and monitor hypertensive outcomes [5]. The study also identified EHRs as a critical component in facilitating mobile health (mHealth) applications, including health coaching interventions (e.g. via calls and text messaging), designed to enhance hypertension management among racial and ethnic minority populations [10–12].

The World Health Organization defines mHealth as medical and public health practices that use mobile devices, such as phones, patient monitors, and personal digital assistants to collect or deliver health data, monitor outcomes, and provide direct care [13]. Due to the urgent need to enhance self-management in primary care, mHealth technologies are sometimes described as the new frontier for promoting healthy lifestyle initiatives [14].

Prior research provides robust evidence supporting their effectiveness, for example in improving medication adherence, increasing the frequency of physical activity, promoting healthy eating, and reducing blood pressure (BP) [15–18]. Importantly, mHealth engagement continues to grow among Black/African American individuals [19]. Although data from a 2014 survey showed that Black/African American and Hispanic/Latino adults were more likely than their White counterparts to use smartphones to retrieve health education content (73% versus 67% versus 58%) [19], this trend likely persists or has even increased today.

However, research on the integrated use of EHRs and mHealth interventions remains in an early stage, revealing two significant knowledge gaps. First, to date, there is no comprehensive systematic review explicitly examining EHR-integrated mHealth interventions aimed at improving hypertension management specifically within Black/African American populations. Second, limited evidence exists on how mHealth interventions delivered in primary care settings can effectively leverage EHR functionalities to enhance hypertension management in Black/African American populations.

This systematic review aims to address these gaps by synthesizing the existing research on EHR-integrated mHealth approaches in primary care settings to improve hypertension management. The specific objectives are to (i) describe the social and clinical characteristics of Black/African American populations represented in EHR-mHealth interventions in primary for managing hypertension and (ii) identify specific EHR-mHealth approaches in primary care and their roles in hypertension management interventions for Black/African American populations.

## METHODS

The manuscript is structured following the Preferred Reporting Items for Systematic Reviews and Meta-analysis guidelines [20]. The systematic review was not registered and made no use of automation systems.

### Search strategy and selection process

Eligible studies were identified via PubMed, Web of Science (WES), and Scopus databases (Table 1). Manual searches were also conducted in Google Scholar to identify relevant studies. References of related systematic reviews and included studies were screened for additional studies. Articles identified via the combined databases were first screened using their titles and abstracts. Then, a full-text review was conducted for those studies that passed the title and abstract screening (Screened by authors E.A and E.G). Catchii, an online program designed to assist with systematic reviews, was used to manage the screening process [21]. The final list of included studies was exported to Zotero citation management program.

**Table 1:** PICO search terms

PICO Element	Keywords
Outcome	Hypertension^*^ OR ‘Blood pressure’ OR BP OR ‘Cardiovascular health’ OR ‘cardiovascular disease’ OR ‘heart health’ OR ‘heart disease’ OR SBP OR DBP OR ‘systolic blood pressure’ OR systolic OR ‘diastolic blood pressure’ OR diastolic or ‘ambulatory blood pressure’ AND
Setting	‘Primary care’ OR ‘family medicine’ OR ‘community health’ OR clinic OR ‘outpatients care’ OR ‘Preventive Medicine’ OR ‘primary health care’ OR CHC OR ‘community health center’ OR emergency AND
Intervention	mHealth OR “m-Health “OR mobile health OR mobile OR digital OR technology OR device OR ‘mobile phone’ OR smartphone OR ‘monitoring device’ OR telemedicine OR telehealth OR PDA OR phone OR ‘text messaging’ OR ‘cellular phone’ OR handheld OR wearable OR application OR android or iphone OR APP OR tablet OR ipad OR ‘smart watch’ AND
Intervention	EHR OR ‘routine data’ OR ‘electronic health’ OR electronic OR ‘e-health’ OR ‘Electronic health record’ OR EMR OR ‘personal health’ OR ‘personal health record’ OR PHR OR ‘electronic medical record’ OR ‘digital health’ OR ‘artificial intelligence’ AND
Population	Black^*^ OR ‘African American’ OR ‘Black or African American’ OR ‘Black/African American’

### Eligibility criteria

Articles were included in this review if they met four criteria: (i) a primary or secondary study outcome focusing on hypertension management or BP control, (ii) study population is primarily Black/African American or included as a majority or second majority population, as part of a multiethnic population, (iii) study was conducted in a primary care setting, and (iv) study provides information on EHR-integration with an mHealth platform (Tables 1 and 2). Furthermore, all database searches were restricted to US-based studies and English-language publications. No year restrictions were applied. The review also excluded conference abstracts with no attached or available peer-reviewed publications. Lastly, the study included all study designs, synthesizing both randomized control trials (RCTs) and non-randomized control trials (non-RCT). This approach ensured we captured enough articles to synthesize and to support a comprehensive overview of EHR–mHealth strategies and how they are implemented in primary care.

**Table 2:** Exclusion and inclusion criteria

PICO		Exclusion criteria	Inclusion criteria
Outcome	Hypertension	Exclude studies with no mention of hypertension management or blood pressure outcomes	
Intervention	Mobile health	Exclude studies with no mention of a form of mobile health (e.g. mobile phones, patient monitoring devices, personal digital assistants (PDAs), and other devices Exclude studies with no mention of a form of EHR-integration	Include studies that contain mobile phones, patient monitoring devices, personal digital assistants (PDAs), telehealth, wearables e.t.c. Include studies with evidence of EHR and mobile integration.
Setting	Primary care	Exclude studies with no mention of primary care settings or community health	Include studies with evidence of primary care settings if not explicitly stated
Population	Black/African American	Exclude studies with no mention of Black/African American populations Exclude studies if Black/African American populations are not the majority or second majority	Include studies even if Black/African American population is not the majority of population under study.

### Data extraction and synthesis

We extracted the following information from each included study: study characteristics relating to the author and year of publication, study aims, study location (city and state), study design, participant social and clinical characteristics, mHealth intervention type, EHR integration, and primary and secondary study outcomes (Tables 1-4; Supplementary files SS-1 and SS-2).

### Risk of bias assessment

The Revised Cochrane risk-of-bias tool for randomized control trials (RoB 2.0) and non-randomized control (ROBINS-I) trials were used to assess the quality of included studies [22]. Individual studies were judged either on a ‘low risk,’ ‘some concerns,’ or ‘high risk’ of bias criteria.

### Ethical compliance

Not applicable.

## RESULTS

### Study selection

**Figure 1:**
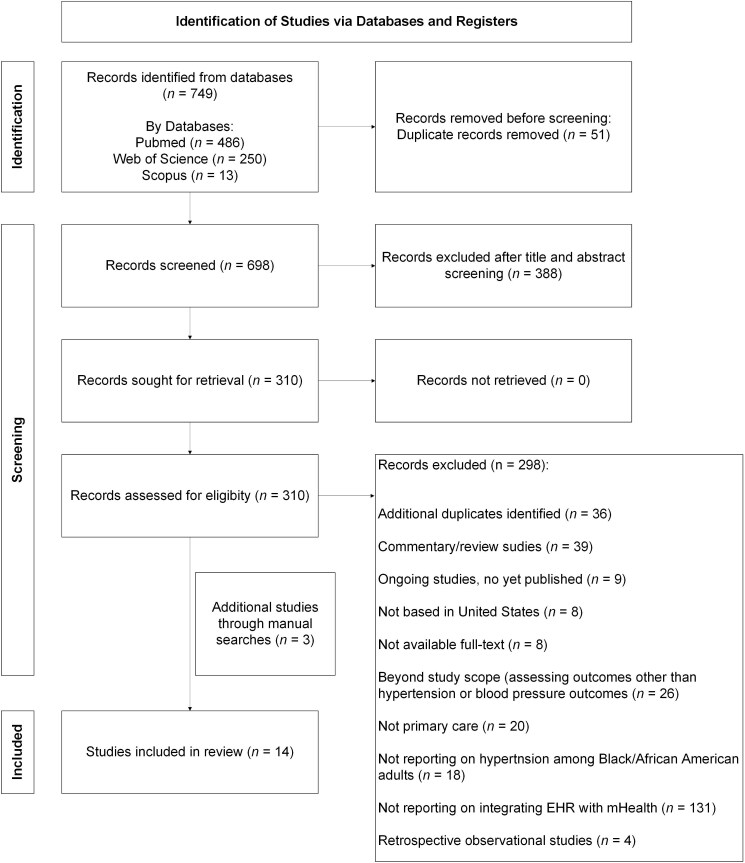
Flow chart.

Searches on the PubMed (*n* = 486), WES (*n* = 250), and Scopus (*n* = 12) databases yielded 749 records (Fig. 1). A total of 698 articles were available for screening after removing 51 duplicates. Then, a combined title and abstract screening removed 388 records. Afterward, a full-text screening was conducted for the remaining 310 articles. Of these, 298 records were excluded if the study was: an additional duplicate (*n* = 36), a commentary or review (*n* = 39), an ongoing study not yet published (*n* = 9), not based in the US (*n* = 8), the full-text could not be retrieved or were poster presentations (*n* = 8), beyond the scope of the review (*n* = 26) i.e. assessing health outcomes other than hypertension or BP, not conducted in a primary care setting (*n* = 20), not reporting on hypertension management in Black/African American populations (*n* = 18), not reporting on EHR integration with mHealth interventions (*n* = 131), and retrospective observational study (*n* = 4). A total of 14 studies were included for synthesis (Fig. 1).

### Assessment of study quality

This review included two study designs: 10 RCTs [12, 23–31], and 4 non-RCTs [14, 32–34]. All the non-RCTs were assessed to be of serious risk of bias, primarily due to the presence of unaddressed confounders which may have influenced behavioral components of the interventions. For example, increased mHealth usability or BP screening rates due to participants awareness that they are taking part in a BP management study. For the RCTs, one study was assessed to be at high risk of bias due to a lack of information on the randomization process and the presence of unaddressed confounders [25]. Further, four additional RCTs were assessed as ‘some concerns,’ resulting from limited information on their randomization process and unaddressed confounders (Supplementary file SS-3) [23, 24, 30, 31].

### Participants social and clinical demographic characteristics

There were 1063 Black/African American individuals included out of 1637 patients across all studies, with a mean cohort age of 52.9 years (Table 3). Overall, across all studies, women comprised most of the study population (68.2%; *n* = 1054), compared to men (31.8%; *n* = 491; note: not all studies stratified the population cohort by gender). Furthermore, diabetes was the most prevalent comorbid health condition reported (*n* = 223; 35.1%), followed by overweight/obesity (20.6%; *n* = 131) and depression (15.1%; *n* = 96; Table 4).

**Table 3:** Black/African American population characteristics

Author	Location	Overall (Ov) (1063/1637)	Age, Mean	Male^a^ (*n* = 491)	Female^a^ (*n* = 1054)
Bennett *et al.* (2018) [23]	North Carolina	Ov: 182/351 In: 93/176 Co: 89/175	50.7	Ov: 112/351 In: 56/176 Co: 56/175	Ov: 239/351 In: 120/176 Co: 119/175
Brewer *et al*. (2023) [14]	Minnesota	Ov: 16	52.6	Ov: 4/16	Ov: 12/16
Davidson *et al.* (2015) [24]	South Carolina	Ov: 18/38 In: 8/18 Co: 10/18	54.3	Ov: 3/38 In: 1/8 Co: 2/10	Ov: 15/38 In: 7/8 Co: 8/10
Eberly *et al.* (2022) [25]	Pennsylvania	Ov: 20/20 In: 10/20 Co: 10/20	55.0	In: 6/10 Co: 4/10	In: 4/10 Co: 6/10
Ferdinand e*t al*. (2023) [32]	Louisiana	32/36	58.7	Ov: 12/36	Ov: 23/36
Idris *et al.* (2022) [26]	Georgia	Ov: 132/132 In: 61/132 Co: 71/132	55.2	Ov: 39/132	Ov: 93/132
Lewey *et al*. (2022) [27]	Pennsylvania	Ov: 70/127 In: 34/70 Co: 36/70	32.2	-	Ov: 70/127 In: 34/70 Co: 36/70
Mehta *et al*. (2019) [29]	Pennsylvania	Ov: 132/149 In [1]: 55/132 In [2]: 46/132 Co: 31/132	53.1	Ov: 56/132 In [1]: 26/132 In [2]: 20/132 Co: 10/132	In [1]: 35/132 In [2]: 34/132 Co: 24/132
Mehta *et al*. 2024 [28]	Pennsylvania	Ov: 223/246 In [1]: 92/223 In [2]: 83/223 Co: 48/223	50.9	Ov: 71/132 In [1]: 26/132 In [2]: 32/132 Co: 13/132	In [1]: 74/132 In [2]: 65/132 Co: 36/132
Naqvi *et al.* (2022) [30]	New York	Ov: 16/50 In: 6/25 Co: 10/25	64.3	Ov: 32/50 In: 16/25 Co: 16/25	Ov: 18/50 In: 9/25 Co: 9/25
Persell *et al*. (2020) [12]	Illinois	Ov: 103/297 In: 56/144 Co: 47/153	58.9	Ov: 115/297 In: 53/144 Co: 62/153	Ov: 182/297 In: 91/144 Co: 91/153
Welch *et al.* (2015) [33]	Massachusetts	Ov: 22/30	60.6	Ov: 13/30	17/30
Schrauben *et al*. (2024) [31]	Pennsylvania	Ov: 21/47 In: 15/23 Co: 6/24	63.0	Ov: 24/47 In: 11/23 Co: 13/24	Ov: 23/47 In: 12/23 Co: 11/24
Zhang *et al.* (2024) [34]	Massachusetts	Ov: 84/98	30.5	-	84/98

Ov: Overall Cohort; In: Intervention group; Co: Control group

a Not all studies stratify patient’s demographics by gender for race. Those studies reported gender count in the overall cohort

**Table 4:** Health comorbidities

Comorbidities	*n* = 635	%	Author, Year
Diabetes	223	35.1	Bennett *et al*. (2018) [23]; Eberly *et al*. (2022) [25]; Ferdinand *et al*. (2023) [32]; Lewey *et al*. (2022) [27]; Mehta *et al.* (2019) [29]; Mehta *et al*. (2024); Persell *et al*. (2020) [12]; Welch *et al*. (2015) [33]; Schrauben *et al*. (2024) [31]
Overweight/Obesity	131	20.6	Bennett *et al.* (2018) [23]; Eberly *et al.* (2022) [25]; Ferdinand *et al.* (2023) [32]; Zhang *et al*. (2024) [34]
Depression	96	15.1	Bennett *et al*. (2018) [23]; Lewey *et al*. (2022) [27]
Chronic Obstructive Pulmonary Disease	56	8.8	Persell *et al*. (2020) [12]
Hyperlipidemia	45	7.1	Bennett *et al.* (2018) [23]
Chronic Kidney Disease	32	5.0	Eberly *et al*. (2022) [25]; Mehta *et al*. (2019) [29]; Mehta *et al.* (2024)[28]; Schrauben *et al*. (2024) [31]
Coronary Artery Disease	20	3.1	Eberly *et al.* (2022) [25]
Heart Failure	12	1.9	Eberly *et al.* (2022) [25]; Persell *et al*. (2020) [12]
Stroke	10	1.6	Naqvi *et al.* (2022) [30]; Persell *et al.* (2020) [12]
Arrhythmias	5	0.8	Eberly *et al*. (2022) [25]
Cardiomyopathy	5	0.8	Eberly *et al*. (2022) [25]

Finally, four studies focused solely on Black/African American patient populations [14, 24–26]. Of the 10 remaining studies that included Black/African American patients as part of a multi-racial and ethnic populations, eight studies included them as a majority [23, 27–30, 32–34], and two studies included them as a second majority [14, 31].

### EHR-integrated mHealth approaches

Eight studies included interactive voice and text messaging (IVT) messaging components in their intervention [23–25, 27–29, 31, 32], and all studies incorporated health monitoring devices. All 14 studies provided participants with BP monitoring cuffs (Supplementary file SS-1). Two of those, in addition, utilized wearable activity trackers, such as Fitbit [27, 31]. One study used body weight scales [23], another included, blood glucose monitors [33], and three studies included electronic pill bottles and medication devices [24, 29, 33]. Finally, three studies included web-based patient portals [23, 25, 26], and 10 studies incorporated smartphone and tablet-based health applications [12, 14, 24–27, 30–32, 34].

**Figure 2:**
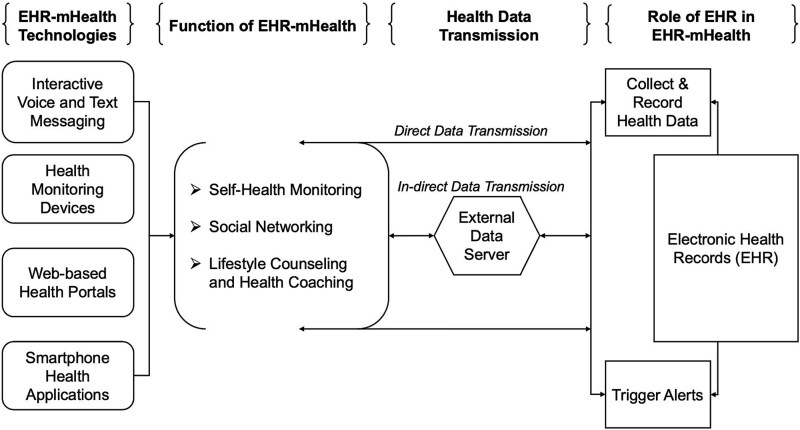
EHR-integrated mHealth approaches for improving hypertension management in Black/African American individuals.

The identified mHealth approaches were implemented for (i) health monitoring or health data collection, (ii) social networking, and (iii) lifestyle counseling or health coaching (Supplementary file SS-1; Fig. 2). For health monitoring, all studies used wireless BP monitor cuffs to collect BP readings. In addition, some studies used electronic medication devices to track medication use and adherence [24, 29, 33], and one study used a cellular network-connected body weight scale to track weight loss progress [23]. Two studies also required some participant groups to manually send BP readings via text messages [25, 31].

Three studies enabled social networking features within their smartphone health applications to support intervention engagement [14, 26, 27]. The Lewey *et al.* [27] study, for example, leveraged gamification features where participants, organized into teams, competed for medals based on highest step count. The study also included social incentives where team members held each other accountable to reach their daily step goals.

Finally, all studies used text messaging and video services via smartphones, tablets, and web applications to deliver lifestyle counseling and health education content. Further, seven studies mentioned using text messaging to send reminders for checking BP, taking and refilling medications, transmitting health data, and collecting feedback on intervention components [24, 25, 27–29, 31, 32].

The included studies used the EHR to collect or record health data and to trigger health alerts. In terms of collecting and recording health data, five studies directly linked their mHealth platforms to the EHR [23, 26, 28, 30, 34]. The second pathway was indirect EHR linkage. Nine studies enabled health data transmission from their mHealth platforms to an external server which then transmitted those data to the EHR [12, 23–25, 27, 29, 31–33]. Some reported server types were data depositories [12, 24, 25] and a cloud platform [32]. Two studies used the EHR to trigger health alerts [28, 34]. For example, Zhang *et al*. [34] programmed the EHR to alert primary care physicians when patients BP readings exceeded 155 mmHg systolic BP or 90 mmHg diastolic BP.

### EHR–mHealth intervention efficacy

Of the 14 included studies, eight successfully achieved their study aims but differed in their outcomes (Supplementary file SS-2). Four studies focused primarily on mHealth uptake and feasibility [25, 26, 31, 34], and four studies focused on BP changes [24, 27, 30, 32].

#### mHealth uptake and feasibility

The Schrauben *et al.* [31] study which compared BP reporting via text messaging to a control group of automatic BP transmission showed a 91% mHealth adoption rate across study groups. In addition, the mHealth usability score was higher than average at 69.3, but no significant differences were observed between the intervention and control group. Likewise, Eberly *et al*. [25] compared acceptability of BP monitoring via text messaging to an online patient portal. All participants in the text messaging group sent BP measurements compared to just 30% in the patient portal group.

#### BP changes

The telehealth and home BP monitoring intervention by Naqvi *et al.* [30] led to a 60% increase in systolic BP control rates in Black/African American patients, compared to 15% in the control group, and 39% among Hispanic patients. Similarly, Davidson *et al.* [24] medication adherence and text messaging health coaching intervention resulted in at least 40% higher systolic BP and diastolic BP over the control groups across all time points. Ferdinand *et al*. [32] text messaging and medication use intervention, resulted in a 10.5 mm Hg reduction in systolic BP and improved medication adherence composite score (2.19 to 1.58). Lewey *et al.* [27] study, which integrated gamification and social networking, successfully increased mean daily step counts over the control group (6175 vs 6042 steps), reduced systolic BP (120.4 vs 123.8 mm Hg), and reduced diastolic BP (80.0 vs 82.3 mm Hg).

## DISCUSSION

This review was conducted to (i) describe the social and clinical characteristics of Black/African American populations represented in EHR–mHealth interventions in primary for managing hypertension and (ii) identify specific EHR–mHealth approaches in primary care and their roles in hypertension management interventions for Black/African American populations.

### Social and clinical characteristics

Middle-aged (52.9 years) Black/African American individuals are most represented in EHR-mHealth interventions for hypertension management in primary care. Indeed, only two studies in our review had a population mean age younger than 35 years. The middle age group as a critical junction in the life course and a predictor of adverse CVD comorbidities in older age may explain their greater representation in hypertension management interventions [35]. A 2018 Coronary Artery Risk Development in Young Adults study revealed that Black/African American adults had the highest cumulative incidence of hypertension by age 55 years; Black/African American women had the highest incidence at 75.7% compared to 75.5% in Black/African American men [35].

It is noteworthy that current interventions have failed to acknowledge the influential role of suboptimal health outcomes in young Black/African American adults and how they contribute to adverse outcomes in middle age. Current trends suggest that younger populations avoid use of primary care services, which contributes to the underdiagnosis of cardiovascular health conditions [36]. Future EHR-mHealth implementation work should focus on hypertension management in younger Black/African individuals, intervening earlier in the life course; and should consider primary care engagement components in their interventions.

The studies in this review reflect strong EHR–mHealth intervention outcomes and findings for Black/African American women as they accounted for a larger proportion of individuals across study samples. One prior study reported that the need to be more knowledgeable about their health condition and assistance with disease management were common facilitators of mHealth use in Black/African American women [37]. The study also revealed that about 64% of women would readily participate in mHealth interventions that used wearable devices and 59% for interventions that used health applications alone. An updated study showed similar results for Black/African American men [38]. Currently, research has not fully examined whether differences exist in mHealth adoption rates between Black/African women and their male counterparts.

The review further revealed that diabetes and obesity were prevalent co-occurring hypertension comorbidities. This is concerning as Black/African American individuals continue to experience 49.6% higher prevalence of obesity and 12.9% higher prevalence of diabetes than other racial and ethnic minority groups [39, 40], which contributes to their hypertension burden. Obesity, in particular, is fast becoming a growing comorbidity concern within Black/African American communities and is strongly associated with both diabetes and hypertension [41]. Inadequate access to quality care, physical inactivity, unhealthy diet, structural and interpersonal discrimination, and stigma, are major factors driving this comorbidity [42]. Despite extensive research, these factors continue to be prevalent. As evidenced in this review, the integration of mHealth and EHR in primary care interventions offers a pathway to address obesity and hypertension outcomes. Interventions leveraging these strategies are unique in that they support self-management or self-monitoring and improve collaborative care between patients and their physicians.

It is equally noteworthy to highlight that persistent systemic inequities will continue to hinder the speed and spread of mHealth adoption in the Black/African American population. Prior research demonstrates that lower-income, rurality, inadequate insurance, poor health literacy, limited technology infrastructure and access, and inadequate support from providers significantly reduces their likelihood of mHealth adoption and sustenance [43, 44], highlighting areas in need of special attention and tailored mitigation efforts.

### EHR-integrated mHealth platforms

Our review demonstrates the combined use of interactive messaging with health monitoring devices, and smartphone health applications, which are common EHR-integrated mHealth approaches in primary care. These platforms were used mainly for health monitoring, social networking, and health coaching.

Text messaging, particularly IVTs, was a major component of these EHR–mHealth interventions. In IVTs, participants would receive text or call reminders to take their medication, check and transmit BP readings, and to initiate follow-up calls or communication with primary care physicians. Studies also implemented IVTs to encourage participants and to provide feedback after they completed intervention activities [23–25, 27–29, 31, 32]. In some cases, primary care physicians would trigger IVTs based on BP readings observed in the EHR to ensure that participants took their medications and measured their BP correctly.

Expansive and targeted implementation of text messaging to improve outcomes is common and highly accepted by Black/African American individuals [37, 45], even in primary care settings. Text messaging is uniquely suited for this role. It is cost-effective, easily accessible, drives personalized communication, and individuals can engage with health-promoting interventions at their convenience [46]. Interestingly, Black/African American individuals, particularly those with chronic health comorbidities, cite these attributes as crucial for enhancing and maintaining their self-care routines [47].

We further found that smartphone health apps are common in mHealth strategies for improving hypertension in Black/African American individuals. Smartphone health applications enhance hypertension self-management in that they provide on-demand access to health data (e.g. BP trends). This accessibility feature provides a platform where users can become more aware of their own bodies and health conditions, and how lifestyle choices impact their health readings [48]. These benefits, including the need to address the impact of race-based discrimination in healthcare, have fostered the rapid development of health applications tailored for Black/African American individuals. However, research is still limited on the best practices for integrating these health applications into the clinical workflow for hypertension management in primary care.

Social networking/interaction is another important yet understudied aspect of smartphone health applications for hypertension management in Black/African American populations. Three studies in the present review incorporated online discussion boards and group competitions where participants exchanged healthy lifestyle ideas and competed for physical activity progress awards [14, 26, 27].

These studies, however, were limited in that they did not report on why social features were included in the interventions. Prior investigations noted that the adoption of social networking in mHealth may be driven by people’s need to share and communicate information with others whom they closely identify with [49]. One qualitative study involving Black/African American adults emphasized that the strong social support they received from relatives and close friends inspired them to live a healthy lifestyle [49]. This need fosters competition which may increase the likelihood of sustained behavior change, even among those who are not interested in engaging in health-promoting behaviors.

Research is still limited on the extent through which ‘social competition and social sharing’ resonates with Black/African American individuals with hypertension, warranting additional research. Future research should also explore primary care physicians’ perspectives on leveraging social networking in mHealth to enhance hypertension management in this population and how clinical guidelines can incorporate both social networking components. An additional avenue for future studies is to assess whether differences exist in hypertension control between EHR–mHealth and non-EHR–mHealth integrated interventions.

### Strengths and limitations

To our knowledge, this is the first review exploring EHR-driven mHealth interventions in primary care settings for hypertension management in Black/African American individuals. It is also unique in that it explored the social and clinical characteristics of Black/African American individuals currently represented in EHR–mHealth interventions. The study also identified existing EHR–mHealth strategies and areas in need of further investigation.

A key limitation of this study is the inclusion of all study designs and not restricting to RCTs. This was done to provide more comprehensive overview of EHR–mHealth strategies and how they are being implemented. Although most of the synthesized studies were RCTs, the inclusion of non-RCTs increased the total number of available studies and the depth of findings. The inclusion of non-RCTs also increased the variations in the reported study aims and outcomes, making it difficult to evaluate their effectiveness.

Furthermore, since the review focuses on US primary care settings, the results and conclusions may not be generalizable to other settings or international regions. Finally, studies were not restricted to those that focused solely on Black/African American patients; the review also included those studies that had Black/African American populations as a majority or second majority, as part of a multiethnic group. Their inclusion presents the risk of overestimating the populations represented in the interventions as well as their effectiveness.

Lastly, the lack of a meta-analysis limits the interpretation of EHR-mHealth effectiveness. We were unable to conduct a meta-analysis due to large variations in the assessment of hypertension outcomes, even among the RCT-only studies. The observed variations included differences in study period (e.g. over 3-months, 6-months, or 9-months), outcome measures (e.g. BP changes, mHealth adoption rates, and physical activity levels), and study population (e.g. Black/African American only, Black/African American as a majority population). Given these variations and a final sample of only 14 studies, the number of comparable articles was insufficient to conduct a robust meta-analysis. Despite these limitations, there is high confidence in the study results, interpretation, and conclusion.

## CONCLUSION

Primary care settings are leveraging mHealth platforms to improve hypertension management in their Black/African American patients. mHealth technologies provide a platform for continuous health monitoring, strengthening collaborative care, and supporting self-management by allowing individuals to take charge of their own health. Specific integration strategies include incorporating EHR–mHealth platforms as part of a hypertension management plan, providing continuous provider and patients training on EHR–mHealth use, and ensuring interoperability between the EHR and mHealth platforms.

## Supplementary Material

SS_1_oqaf029

SS_2_oqaf029

SS_3_oqaf029

## Data Availability

The underlying data, consisting of all identified and screened articles (included and excluded), will be shared on reasonable request to the corresponding author.
