# Supplementary material for: Electronic health records-integrated mobile health interventions in primary care to improve hypertension management in Black/African American populations: a systematic review
Source: Oxf Open Digit Health. 2025 Nov 3;3:oqaf029. doi: 10.1093/oodh/oqaf029 (PMC12704441; doi:10.1093/oodh/oqaf029)
Supplement: SS_1_oqaf029 [file ss_1_oqaf029.docx]

| SS1. Summary of EHR-integrated mHealth interventions in primary care to improve hypertension management in Black/African American populations | | | | | |
| --- | --- | --- | --- | --- | --- |
| Author, Year | Aim | Overall Intervention | EHR-mHealth Platform | Role of EHR-mHealth | Integration of EHR with mHealth |
| Bennett et al. 2018 | Reduce body weight; Improve BP, fasting lipids, glucose, and HbA1c. | Weight loss counseling with Dietician and PCP;  Weekly BP monitoring;  BP monitoring voice and text reminders. | Interactive text messaging and calls; Weight scale with cellular network;  Web-based application;  BP monitor cuff. | BP Self-monitoring; Weight loss counselling and health coaching. | EHR connected to weight scale to collect health data. |
| Brewer et al. 2023 | Feasibility of mHealth integration;  Improve BP. | 10-module education series;  3-days-weekly BP monitoring;  Weekly CHW-calls. | Smartphone health application (FAITH!);  BP monitor cuff with Bluetooth and cellular network. | BP Self-monitoring; Social networking; Interactive self-assessment. | Smartphone application connected to EHR-linked activity program to collect/transmit health data. |
| Davidson et al. 2015 | Reduce BP; Increase medication adherence. | Daily BP monitoring;  BP monitoring text reminders;  Medication use text/call reminders. | Electronic medication device with cellular network;  Text messaging; Smartphone health application. | BP self-monitoring; Tracking medication use. | Smartphone application connected to EHR-linked server to collect/transmit health data. |
| Eberly et al. 2022 | Increase BP screening. | 2x-daily BP monitoring;  Daily BP text reminders & automated reply. | Smartphone health application;  Web-based patient portal;  BP monitor cuff; Interactive text messaging. | BP self-monitoring; Transmit health data; Health monitoring reminders. | Smart phone application connected to EHR-linked server to collect/transmit health data;  EHR linked to patient portal. |
| Ferdinand et. al. 2023 | Increase adherence to hypertension medication use; Improve SBP and DBP | 2x-daily BP monitoring;  Daily medication use via text/call reminders; | BP monitor cuff with Bluetooth; Smartphone health application (Sphygmo); Bidirectional electronic messaging. | BP self-monitoring; Medication use reminders; Motivational messaging. | Smartphone health application connected to EHR-linked server to collect/transmit health data. |
| Idris et al. 2022 | Explore the determinants of mHealth non usage attrition | Weekly meeting with health coach. | Web and Smartphone health application (Health360x). | Social networking; Health education content;  Lifestyle monitoring. | Health application linked to the EHR to collect/transmit health data. |
| Lewey et al. 2022 | Improve physical activity | Daily motivational text messaging;  Daily BP monitoring. | Activity tracker (Fitbit);  BP monitor cuff; Interactive text messaging; Smartphone health application (Fitbit health). | Step count and distance walked/run tracking;  BP self-monitoring; Gamification & Social networking; Motivational text messages. | Smartphone health application connected to EHR-linked server. |
| Mehta et al. 2019 | Improving medication adherence and BP control | Daily medication use via text reminders. | Electronic pill bottles with cellular network; Automated bidirectional text messaging; Cellular phone. | Track Medication Use;  Motivational text messages. | Electronic pill bottles connected to the EHR-linked server (Way to Health) to collect medication use data. |
| Mehta et al. 2024 | Improve BP control | BP monitoring text reminders;  Weekly medication use via text reminders;  Support partners. | BP monitor cuff with Bluetooth; Automated bidirectional text messaging. | BP self-monitoring; Motivational text messages;  Track Medication use;  Lifestyle counseling. | EHR used to send alerts. |
| Naqvi et al. 2022 | Improve SBP and DBP | Nurse-led Bi-weekly health coaching;  PCP and pharmacist visit. | BP monitor cuff with cellular network; Electronic tablet. | BP self-monitoring; Video calls with clinical team. | EHR connected to BP monitor to collect/transmit health data. |
| Persell et al. 2020 | Improve SBP | Daily BP monitoring;  Health app BP monitoring reminders. | BP monitor cuff with Bluetooth; Smartphone health application (Omron); Hypertension coaching app with AI (HTN Pro). | BP self-monitoring; Lifestyle coaching and counseling; Medication reminders;  Track physical activity, sleep, eating habits. | EHR connected to server to collect and transmit data from health applications. |
| Schrauben et al. 2024 | Improve BP and physical activity self-monitoring | 3x weekly BP monitoring;  5x weekly step counts. | BP monitor cuff with Bluetooth;  Activity tracker (Fitbit Inspire II);  Smartphone health application (Fitbit health);  Text messaging. | BP self-monitoring; BP transmission reminders;  Step count transmission reminders;  Lifestyle counseling; Technological assistance. | EHR connected to server to collect/transmit health data;  EHR-linked server connected to health applications and text service. |
| Welch et al. 2015 | Feasibility of telehealth monitoring program | Nurse-led alert monitoring;  Daily fasting BG reading;  3x weekly BP reading;  Daily medication use. | Electronic pill bottles with cellular network;  BG meter with cellular network;  BP monitor cuff with cellular network. | Medication adherence reminders; Pill organization;  BP self-monitoring; Blood glucose monitoring. | EHR connected to server linked to pillbox to collect and transmit data to physicians. |
| Zhang et al. 2024 | Feasibility of home BP monitoring | Daily BP reading;  Nurse-led remote monitoring. | iPad tablet with cellular network;  BP monitor cuff with Bluetooth;  Heath coaching tablet application. | BP self-monitoring; Lifestyle counselling. | EHR connected to iPad to collect/transmit health data;  EHR triggers alerts. |

BP: Blood Pressure, BG: Blood Glucose, SBP: Systolic Blood Pressure, DBP: Diastolic Blood Pressure, PCP: Primary Care Physician, CHW: Community Health Worker, EHR: Electronic Health Records, AI: Artificial Intelligence
