# Supplementary material for: Electronic health records-integrated mobile health interventions in primary care to improve hypertension management in Black/African American populations: a systematic review
Source: Oxf Open Digit Health. 2025 Nov 3;3:oqaf029. doi: 10.1093/oodh/oqaf029 (PMC12704441; doi:10.1093/oodh/oqaf029)
Supplement: SS_2_oqaf029 [file ss_2_oqaf029.docx]

| SS2. Main study characteristics and findings | | | | | |
| --- | --- | --- | --- | --- | --- |
| Author, Year | Design | Intervention arms | Study duration | Outcomes assessment | Outcomes |
| Bennett et al. 2018 | RCT | 2-arms | 12-months | Baseline, 6- and 12-months | 6-months:  SBP: −4.6 (−7.5, −1.7)  DBP: −4.1(−5.9, −2.4)  12-months  SBP: −8.4 (−11.4, −5.3)  DBP: −5.2 (−7.1, −3.3)  *P*-values >0.5 |
| Brewer et al. 2023 | Pre-test/ post-test | 1 | 3-months (10-weeks) | Baseline, 10-weeks | Baseline:  SBP: 149.7 (15.3)  DBP: 93.4 (8.9)  10-wks  SBP: 143.2 (17.0)  DBP: 90.6 (19.9)  *P*-values >0.5 |
| Davidson et al. 2015 | RCT | 2-arms | 6-months | Baseline, 1-, 3-, and 6-months | SBP – int. vs control  Month 1:  70.6% vs 15.8%  Month 3:  94.4% vs 55.0%  Month 6:  94.4% vs 41.2%  *P*<0.05  DBP:  Month 1:  100.0% vs 68.4%  Month 3:  100.0% vs 65.0%  Month 6:  94.4% vs 76.5%  *P*<0.05 |
| Eberly et al. 2022 | RCT | 2-arms | 1-month (28-days) | Baseline, 28-days | More BP readings received from patients in text messaging group (10; 100%) – *P*<.05 |
| Ferdinand et. al. 2023 | Pre-test/ post-test | 1-arm | 2-months (8-weeks) | Baseline, 8-weeks | Baseline  SBP: 142.19  DBP: 81.19  *P*-values for SBP <.05  8-weeks  SBP: 131.69  DBP: 79.49  *P*-values for DBP >.05  Medication adherence:  Baseline mean score:  2.19  8-weeks: 1.58; *P*<.05 |
| Idris et al. 2022 | RCT | 2-arm | 6-months | Baseline, 6-months | Increased mHealth non-usage in health coach group and those 4 years or more of college  *P*<.05 |
| Lewey et al. 2022 | RCT | 2-arms | 3-months (12-weeks) | Baseline, 12-weeks | Baseline:  SBP: 121.4 (12.9)  DBP: 82.2 (11.5)  End of Study:  SBP: 120.4 (11.7)  DBP: 80.0 (12.6) |
| Mehta et al. 2019 | RCT | 3-arms | 4-months | Baseline, 4-months | No significant change in BP  *P*>.016 |
| Mehta et al. 2024 | RCT | 3-arms | 4-months | Baseline, 4-months | No significant improvement in BP |
| Naqvi et al. 2022 | RCT | 2-arms | 3-months (12-weeks) | 12-weeks | Improved SBP control (60%)  *P*<0.01 |
| Persell et al. 2020 | RCT | 2-arms | 6-months | Baseline, 6-months | No significant improvement in BP |
| Schrauben et al. 2024 | RCT | 2-arms | 3-months (12-weeks) | Baseline, 1-week, 6-weeks, 12-weeks | Improved BP reading from baseline (>3 weekly test)  Improved blood glucose control (*P*<0.05) |
| Welch et al. 2015 | Pre-post test | 1 | 3-months | Baseline, 3-months | No significant change in BP  *P*>0.05  Higher mean step count |
| Zhang et al. 2024 | Pre-post test | 1 | 2-months (8-weeks) | Baseline, 7-days, 8-weeks | PPT visits completion rates higher among those who are fully engaged with remote monitoring  *P*<0.05 |

RCT: Randomized Control Trial, SBP: Systolic Blood Pressure, DBP: Diastolic Blood Pressure, BP: Blood Pressure, Int: Intervention, PPT: Postpartum
